# Supplementary material for: Systemic control of immune cell development by integrated carbon dioxide and hypoxia chemosensation in Drosophila
Source: Nat Commun. 2018 Jul 11;9:2679. doi: 10.1038/s41467-018-04990-3 (PMC6041325; doi:10.1038/s41467-018-04990-3)
Supplement: Supplementary file 1 — Supplementary Information [file 41467_2018_4990_MOESM1_ESM.pdf]

Supplementary Information

Systemic control of immune cell development by integrated carbon dioxide and hypoxia chemosensation in *Drosophila*

Cho et al.,

Supplementary Figure 1

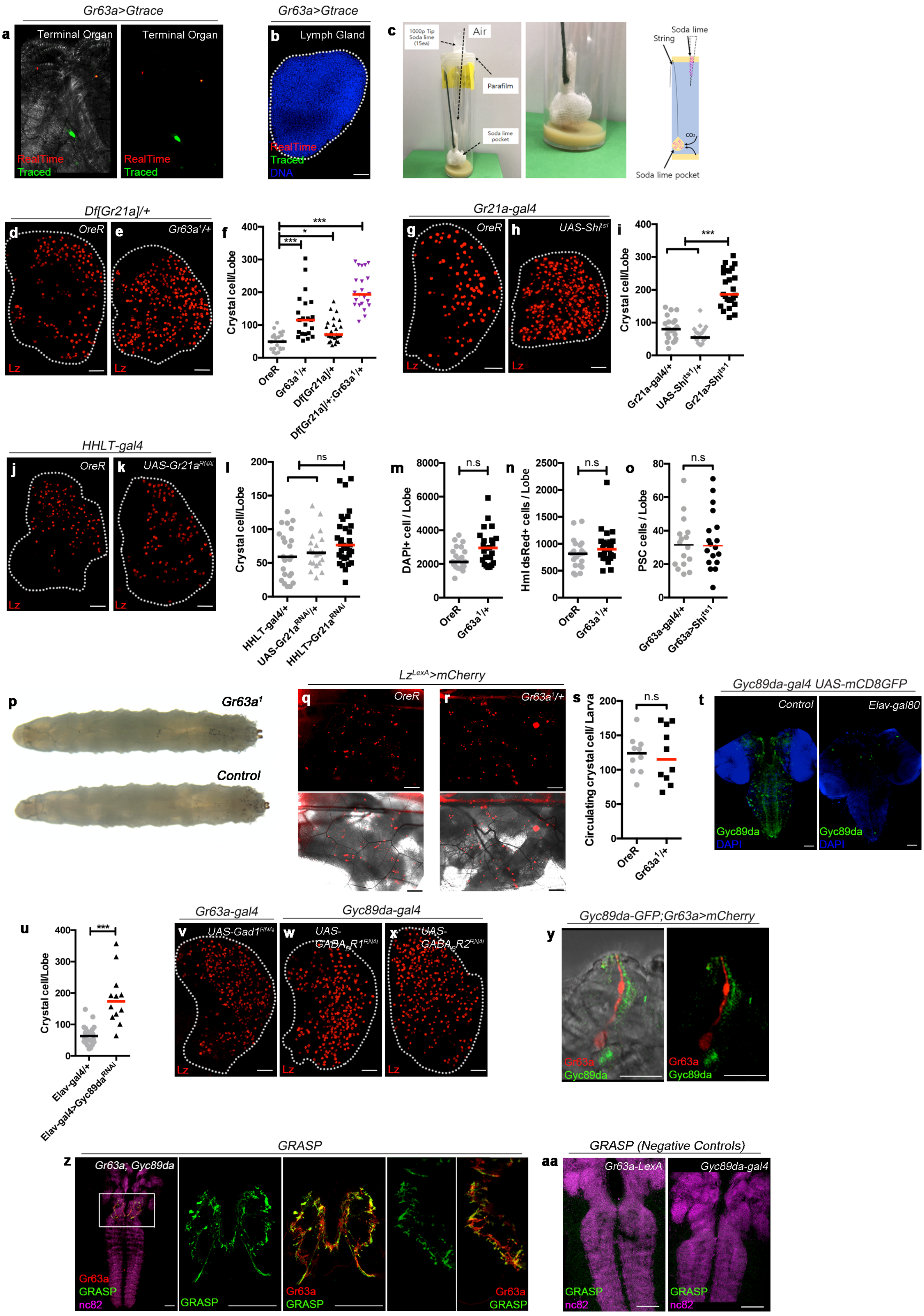

Supplementary Figure 1. CO_2/O_2 chemosensation controls crystal cell differentiation.

Lymph glands are demarcated by white dotted lines. Graphs in **(f), (i)**, **(l)** and **(u)** indicate the number of crystal cells per one lymph gland lobe of the relevant genotypes (n.s, not significant; *$p<0.01$; ***$p<0.0001$). Bars in the graph represent the median. Scale Bar: 20μm, unless otherwise indicated.

a-b. Gr63a is expressed in the neuron.

Lineage-traced expression of *Gr63a-gal4* is seen in the terminal organ (Real-time expression of *Gr63a-gal4* is shown in red; traced expression, in green) **(a)**, but not in the lymph gland (*Gr63a-gal4; UAS-Gtrace*) **(b)**.

c. Schematic diagram of the soda-lime experimental set-up (also see Methods for further details). The soda-lime pocket is hung above the food (soda-lime is indicated in light purple) to scavenge respiratory CO_2 from animals or yeast. Incoming air flowing through the pipette tip containing soda-lime is also scavenged for CO_2.

d-i. Both *Gr63a* and *Gr21a* are involved in the control of crystal cell differentiation.

One copy loss of *Gr21a* (*Df[Gr21a]/+*) is sufficient to increase crystal cell number **(d)**. The trans-heterozygote *Df[Gr21a]/+; $Gr63a^1$/+* further enhances this phenotype **(e)**. Quantitation shown in **(f)**. Synaptic inhibition of CO_2SN increases crystal cell differentiation (*Gr21a-gal4; UAS-Shi^{ts1}*) **(g, h)**. Quantitation shown in **(i)**.

j-l. *Gr21a* is not expressed in the lymph gland, and thus knockdown in that organ has no effect on crystal cell number (*HHLT-gal4; UAS-$Gr21a^{RNAi}$*) **(j, k)**. Quantitation shown in **(l)**.

m-s. Loss of CO_2SN does not alter developmental landscapes of larval blood. Lymph glands from *Gr63a*^{1}*/+* mutants do not change the total cell number **(m)**, *Hml*+ plasmatocytes **(n)**, or *Antp*+ PSC cells **(o)** (*Hml-dsRed; Dome-Meso-GFP; Gr63a*^{1}*/+*). Melanization function of crystal cells in circulation is not perturbed upon loss of CO_2SN **(p)**. Distribution of *Lz*+ crystal cells in the hematopoietic pocket (Lz, red) **(q-r)** or the number of *Lz*+ crystal cells in circulation is not changed when CO_2SN is mutated (*Lz-LexA LexAop-mCherry; Gr63a*^{1}*/+*) **(s)**. Scale Bar: 100μm.

t. *Gyc89da-gal4* expression in the brain is completely suppressed when *Elav-gal80* is included in the genotype (*Gyc89da-gal4; Elav-gal80*). Scale Bar: 50μm.

u. Loss of the receptor *Gyc89da* throughout all neurons increases the number of crystal cells in the lymph gland (*Elav-gal4; UAS-Gyc89da*^{RNAi}). Given its inhibitory functions under atmospheric O_2 concentration, loss of the receptor is thought to activate HypSNs.

v-x. GABA to $GABA_B$ receptor signaling controls the CO_2SN and HypSN interaction.

Corresponding lymph gland images of data presented in **Fig. 2l** and **2m**. Expression of RNA*i* against *Gad1* in the CO_2SN enhances differentiation of crystal cells (*Gr63a-gal4; UAS-Gad1*^{RNAi}) **(v)**. Loss of *GABA*$_B$*R1* **(w)** or *GABA*$_B$*R2* **(x)** in HypSNs also leads to extra crystal cell differentiation.

y. At the level of the terminal organ, CO_2SN and HypSNs run on parallel tracks that do not overlap (*Gr63a-gal4; UAS-mCherry, Gyc89da-GFP*). Scale Bar: 30μm.

z-aa. GRASP expression of the CO_2SN and HypSNs in the SEG.

GRASP signal resulting from points of contact is shown in green (demarcated by white box; magnified images of the boxed area are shown in the following

panels; Scale Bar: 30μm) **(z)**. CO_2SN is marked in red and the overlap with GRASP signal shown in yellow. At high magnification, GRASP signal are suggestive of synapse formation (Full genotype: *Gyc89da-gal4, LexAoP-CD2RFP; Gr63a-LexA, UAS-CD4-spGFP*$_{11}$*, LexAop-CD4-spGFP*$_{1\text{-}10}$). Negative control experiments for the GRASP data shown in **Supplement-Fig. 1z**. *Gr63a-LexA* alone (*Gr63a-LexA; UAS-CD4-spGFP*$_{11}$*, LexAop-CD4-spGFP*$_{1\text{-}10}$) or *Gyc89da-gal4* alone (*Gyc89da-gal4; UAS-CD4-spGFP*$_{11}$*, LexAop-CD4-spGFP*$_{1\text{-}10}$) does not give rise to a GRASP signal (Scale Bar: 50μm) **(aa)**.

Supplementary Figure 2

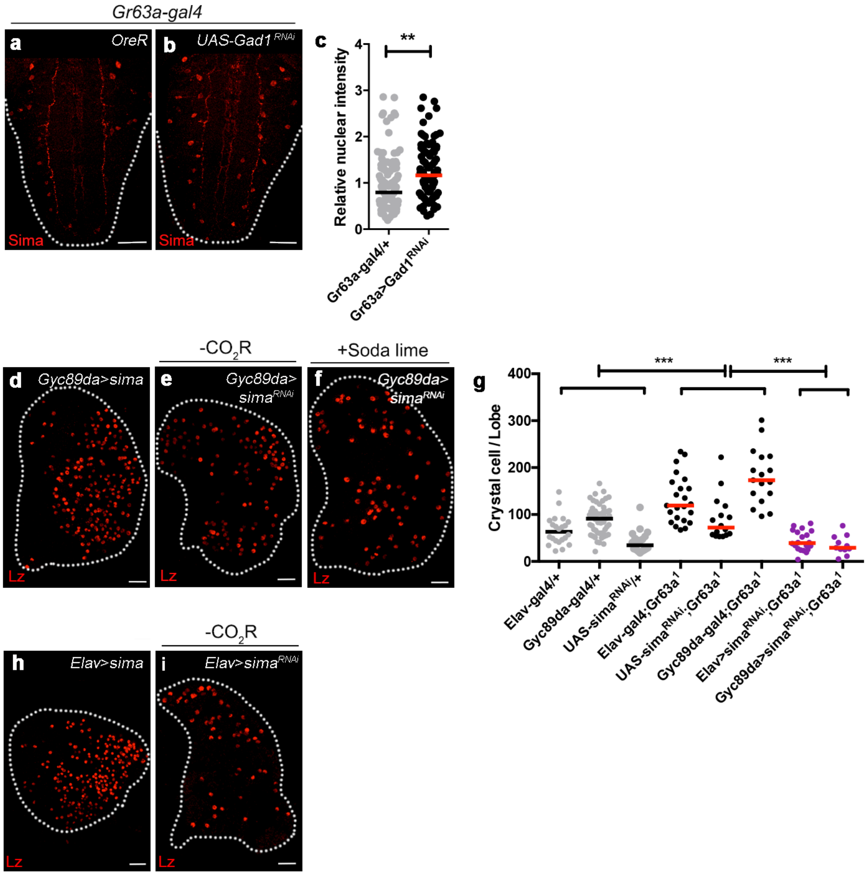

Supplementary Figure 2. Sima expression in HypSNs is downstream of CO_2SN.

Bars in **(c), (g)** indicate the median. (***p*<0.001). Scale Bar: 20μm, unless otherwise indicated.

a-c. Loss of *Gad1* in the CO_2SN leads to accumulatation of Sima in the brain. Compared to controls (*Gr63a-gal4/+*) **(a)**, expression of RNA*i* against *Gad1* in the CO_2SN (*Gr63a-gal4; UAS-Gad1^{RNAi}*) induces Sima expression in the ventral nerve cord **(b)**. Quantitation of relative nuclear Sima staining is shown in **(c)**. Scale Bar: 50μm.

d-i. Sima in the HypSN is essential for the crystal cell expression. Representative lymph gland images corresponding to quantitative data shown in main Fig. 3g **(d)**, Fig. 3h **(e)**, Fig. 3i **(f)**, Fig. 3j **(h)** and Fig. 3k **(i)**. Expression of RNA*i* against *sima* in all neurons (*Elav-gal4 UAS-sima^{RNAi}; Gr63a^1/Gr63a^1*) or in *Gyc89da*-expressing cells (*Gyc89da-gal4 UAS-sima^{RNAi}; Gr63a^1/Gr63a^1*) is sufficient to restore the number of crystal cells induced in CO_2SN mutants **(g)**.

Supplementary Figure 3

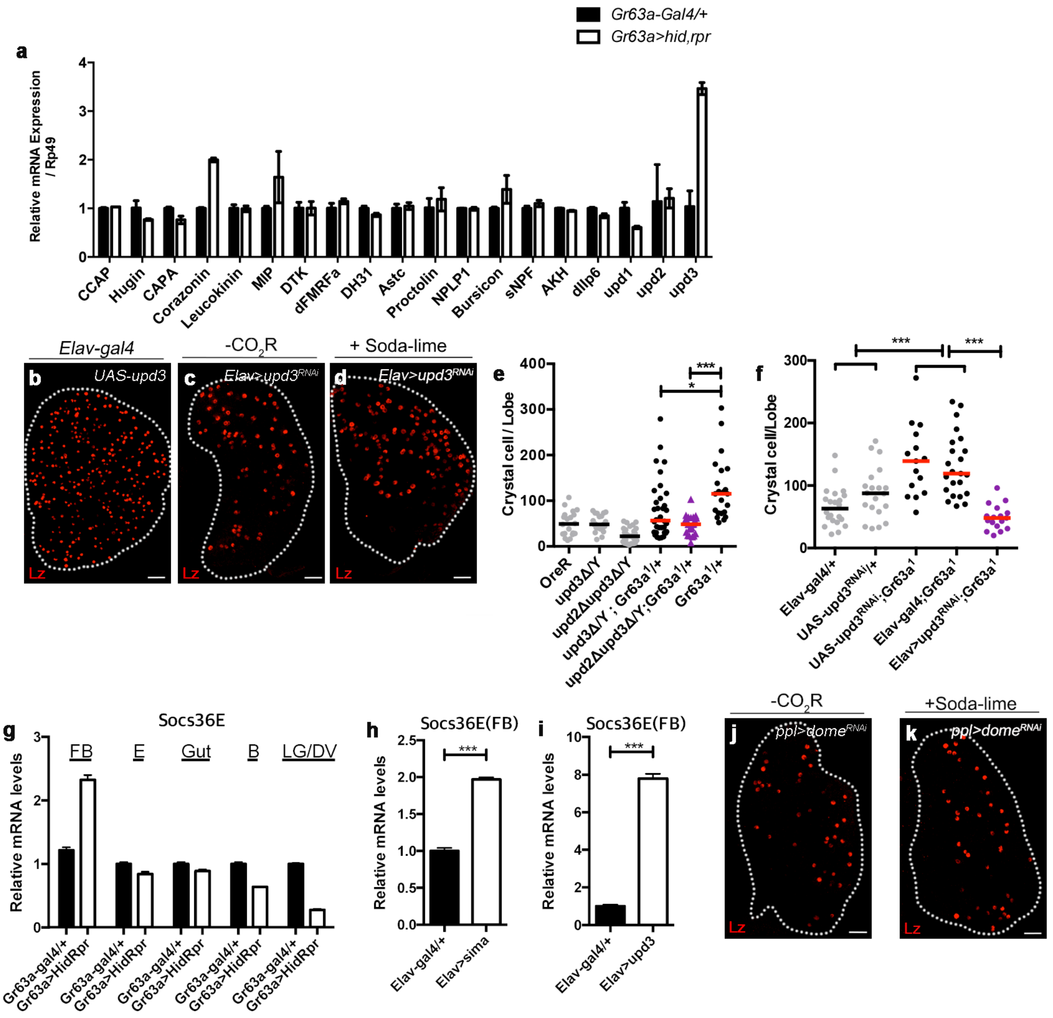

Supplementary Figure 3. Upd3 expressed in the brain is necessary for excess crystal cell differentiation upon loss of CO_2SN.

Error bars in the graph **(a)** and **(g-i)** represent standard deviation (****p*<0.0001). Bars in **(e-f)** indicate the median. Scale Bar: 20μm.

a. A mini-screen identifying Upd3 as a secretable factor in the brain. Transcript levels of *CCAP, Hugin, CAPA, Corazonin, Leucokinin, MIP, DTK, dFMRFa, DH31, Astc, Proctolin, NPLP1, Bursicon, sNPF, AKH, dilp6, upd1, upd2* and *upd3* are tested in the CO_2SN mutant background (*Gr63a-gal4 UAS-hid,rpr*). Genetic manipulation of neither Coraznonin nor MIP gives rise to the crystal cell change while Upd3 does.

b-d. Representative lymph gland images of data shown in Fig. 4c **(b)**, Fig. 4e **(c)** and Fig. 4f **(d)**.

e-f. Simultaneous mutations in *upd3* rescues the CO_2SN mutant phenotype. *Upd3*Δ single mutant partially restores the crystal cell phenotype shown in *Gr63a* mutants (*Upd3Δ/Y; Gr63a^1/+*). *Upd2ΔUpd3Δ* double knock-out mutants completely rescue the phenotype (*Upd2ΔUpd3Δ/Y; Gr63a^1/+*) **(e)**. Expression of RNA*i* against *upd3* in all neurons rescues the number of crystal cells induced in CO_2SN mutants (*Elav-gal4 UAS-upd3^{RNAi}; Gr63a^1/Gr63a^1*) **(f)**.

g. *Socs36E* mRNA up-regulation is limited to the fat body. (FB, fat body; E, epidermis; Gut; B, brain; LG/DV, Lymph gland/Dorsal vessel).

h-i. Expression of *sima* (*Elav-gal4; UAS-sima*) **(h)** or of *upd3* (*Elav-gal4; UAS-upd3*) **(i)** in the brain is sufficient to enhance *Socs36E* transcript levels in the fat body.

j-k. Representative lymph gland images of crystal cell quantitation data shown in Fig. 4r **(j)** and Fig. 4s **(k)**.

Supplementary Figure 4

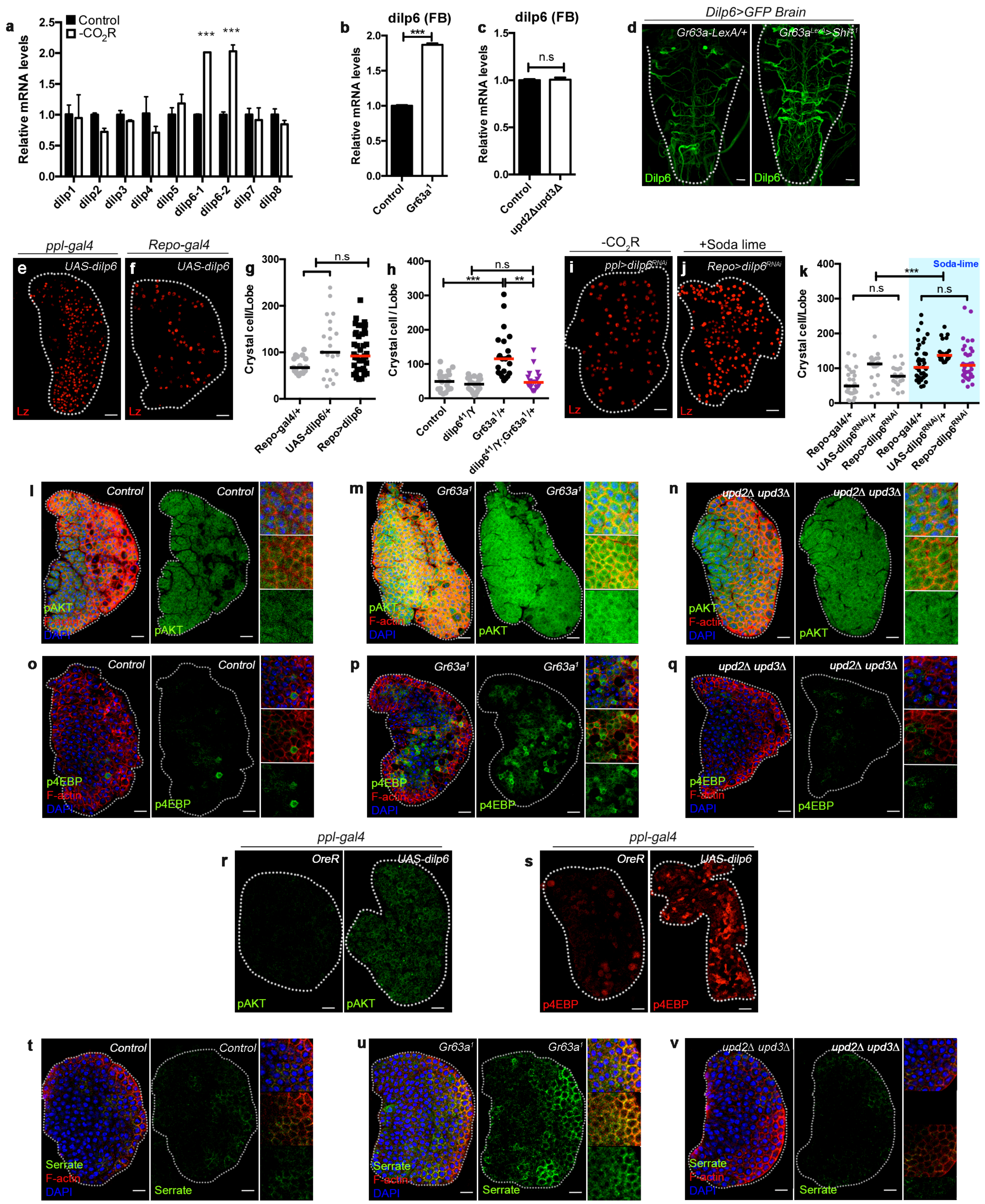

Supplementary Figure 4. CO_2SN/HypSNs systemically control the Dilp6-InR-Serrate cascade

Graphs in **(g-h)** and **(k)** indicate the number of crystal cells per lymph gland lobe of relevant genotypes (n.s, not significant; ***p<0.0001). Error bars in **(a-c)** represent standard deviation. Bars in the graphs **(g-h)** and **(k)** indicate the median. Scale Bar: 20μm. Blue background indicates soda-lime experiments **(k)**.

a. *dilp6* is transcriptionally up-regulated upon loss of CO_2SN (*Gr63a-gal4; UAS-hid,rpr*; whole larvae). Two independent primer sets (1 and 2) were used.

b-c. *dilp6* in the fat body is up-regulated when CO_2SN is mutated (*Gr63a^1*) whereas *upd2Δupd3Δ* mutants do not show any changes.

d. Normal expression of *dilp6* in the brain is indistinguishable from CO_2SN mutants (*Gr63a-LexA, LexAop-Shi^{ts1}; Dilp6-gal4, UAS-GFP*).

e. Representative lymph gland image of data shown in main Fig. 5e.

f-g. Expression of *dilp6* in glia (*Repo-gal4; UAS-dilp6*) does not alter crystal cell differentiation **(f)**. Quantitation is shown in **(g)**.

h. Genetic combination of *Gr63a^1* and *dilp6^{41}* rescues the crystal cell phenotype derived by *Gr63a^1* mutation (*dilp6^{41}/Y; Gr63a^1/+*).

i. *dilp6* knockdown in the fat body recovers the number of crystal cells upon loss of CO_2SN shown in Fig. 5f (*Gr63a-LexA, LexAop-Shi^{ts1}*; *ppl-gal4, UAS-dilp6^{RNAi}*).

j-k. Alteration in *dilp6* expression in the glia does not suppress the soda-lime-mediated phenotype in the lymph gland (*Repo-gal4; UAS-dilp6^{RNAi}*) **(j)**. Quantitation is shown in panel **(k)**.

l-q. Insulin receptor (InR) activation by Dilp6 induces its downstream targets. Expression of pAKT or p4EBP is enhanced in the lymph gland of *Gr63a^1* mutant. Wild-type lymph glands exhibit low pAKT or p4EBP protein expression during

the early third instar **(l and o)** while *Gr63a*^{1} mutant lymph glands promote membrane localization of pAKT or cytoplasmic p4EBP **(m and p)**. *upd2Δupd3Δ* mutants do not show any significant change in pAKT or p4EBP expression **(n and q)**. Magnified images are shown in each figure (pAKT in green; F-actin in red; DAPI in blue).

r-s. Overexpression of *dilp6* in the fat body (*ppl-gal4; UAS-dilp*6) enhances both pAKT **(r)** and p4EBP **(s)**.

t-v. Serrate expression is upregulated in the *Gr63a*^{1} mutant lymph gland. Lymph glands from the late second instar larvae express very low levels of Serrate in wild-type controls **(t)**. In *Gr63a*^{1} mutants, Serrate expression is increased during the same stages **(u)**. This increase is not observed in *upd2Δupd3Δ* mutants **(v)**. Magnified images are shown in each figure (Serrate in green; F-actin in red; DAPI in blue).

Supplementary Figure 5

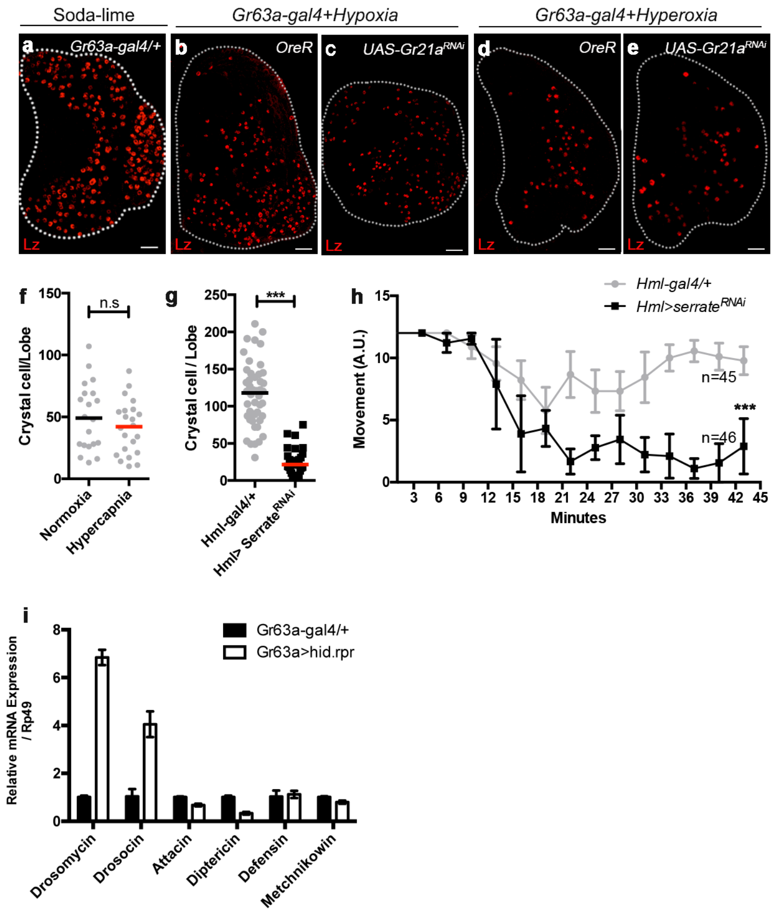

Supplementary Figure 5. Integration of O_2 and CO_2 chemosensory function in the blood.

Graphs in **(f)** and **(g)** indicate the number of crystal cells per lymph gland lobe of relevant genotypes (n.s, not significant; ****p*<0.0001). Error bars in **(i)** represent standard deviation. Bars in graphs **(f)** and **(g)** indicate the median. Error bars in **(h)** indicate standard error of the mean. *p* value for **(h)** is analyzed by two-way ANOVA using each genetic group. Scale Bar: 20μm.

a-e. Representative lymph gland images of crystal cell quantitation data shown in main Fig. 1j **(a;** a repeat of Fig. 1j**)**, Fig. 6c **(b,c)**, and Fig. 6d **(d,e)**.

f. 13 % CO_2 does not alter the crystal cell differentiation in the lymph gland compared to normoxic controls.

g-h. Crystal cell plays a beneficial role during hypoxia.

Knock-down of *Serrate* in the blood significantly reduces the number of crystal cells in the lymph gland (*Hml-gal4 UAS-Serrate^{RNAi}*) **(g)**. Blood development in the larval lymph gland is the second wave of hematopoiesis and is utilized during adult stages under conventional conditions. Flies expressing *Hml-gal4 UAS-Serrate^{RNAi}* exhibit reduced mobility from 12-15 minutes after exposure to 1 % hypoxia and subsequently become paralyzed. Wild-type flies actively move during the same period of time **(h)**.

i. mRNA levels of both *Drosomycin* and *Drosocin* are up-regulated in the fat body when CO_2SN is mutated.

Supplementary Table 1. RNAi efficiencies verified in this study

Target gene	Line	Efficiency
Gr21a	BL31281	75%
dilp6	VDRC102465	94%
upd3	VDRC106869	89%
sima	VDRC106187	75%
domeless	VDRC19717	55%
Gad1	BL51794	52%
$GABA_bR1$	VDRC101440	69%
$GABA_bR2$	BL50608	50%
Serrate	VDRC27172	35%

Supplementary Table 2. Sequences used for qPCR analysis

Target gene	**Forward**	**Reverse**
sima	AACTATCGCGAGGAGTCGAA	CGTTAGCAGGGGCATATCAT
Dilp1	ACAACGGTGCAGCAGTACAT	CCTTGGCAGCGTATTAAAGC
Dilp2	CTCAATCCCCTGCAGTTTGT	CGCAGAGCCTTCATATCACA
Dilp3	CAACGCAATGACCAAGAGAA	TGGCAGCACAATATCTCAGC
Dilp4	GGATGCTAGAGTGCGAGACC	AGCCCTCCTTGCAACACTC
Dilp5	TCAATTCAATGTTCGCCAAA	AGTCGCAGTATGCCCTCAAC
Dilp6-1	ACCCACGGAATACGAACAGA	TGCAAGAGCTCCCTGTAGGT
Dilp6-2	CCCACGGAATACGAACAGAG	GCAAGAGCTCCCTGTAGGTG
Dilp7	AAAAGAGGACGGGCAATGAT	CGCTTGTTGGATGGACAATA
Dilp8	ACATCAGCCGAAGCAGCTAT	CCTCCTCGCACTGGTTTAGA
Rp49	GGCCCAAGATCGTGAAGAAG	ATTTGTGCGACAGCTTAGCATAT C
upd3	TGCCCCGTCTGAATCTCACT	GTGAAGGCGCCCACGTAA
Socs36E	CAGTCAGCAATATGTTGT	ACTTGCAGCATCGTCGCT
domeless	TGACATCGGCTATACCAC	GGCGTTACGTAGCCATTG
Gr21a	GGAACTGGACAAGCACGATT	CAGCACCAGAACAACAATGG
Serrate	GATGGATCGGGTCACAGTCT	CGACTTCTCCTCCTCGTGAC

CCAP	TATCCGCCATTCTCGCTATT	TTTGCCGAAAGATTTCCTTG
Hugin	GGCTTCCTGCTACATCCTTG	TTCTCCTCCAGCTCGTCAAT
CAPA	AGACGGACCACGACAAGAAC	CACCAGACCACTGGCTTTCT
Corazonin	CAACGGCAAGAGGTCCTTTA	ACTCGGTTGGCATTGAAGTC
Leucokinin	GCTGCAGCTCTCCAAGTACC	ACTCCTTGTCACCGAACTGG
MIP	AATTTGGTGGCCAGTGGTAG	TCATGTAGATGTCCGGATCG
DTK	TCAATTCCTTTGTGGGGATG	TGCTCATGATCGTCACCAAT
dFMRFa	GTGCTGCACTTCCAGAAACA	CTGCCATACCGATCCATAGC
DH31	TCCTCCTCTTCTGCCTCTTG	AAGCCAAAGTCCACGGTTC
AstC	GGCCTACTCCTCACCCTGTT	GGGTCTGTATTGGGCAAAGA
Proctolin	CTGATGCTGCAGATTTTGGA	ACCCATGGCACTCAGTTTCT
NPLP1	GATGATGTCGCCAATGTGTC	GAACGTTTGTCCTCCTCCAG
Bursicon	TATCCAGGTTTCGGGCAGTA	GATGCCAGACTCCTCAATGG
sNPF	GTTCCTCAGTTCGAGGCA	TGGAACTCCGACGTATCG
AKH	TGAATCCCACGAGCGAAG	ATCTCGAGCAGCATTTCG
upd1	TCCACACGCACAACTACA	CCAGCGCTTTAGGGCAAT
upd2	AGTGCGGTGAAGCTAAAG	GCCCGTCCCAGATATGAG
Drosomyci	GTACTTGTTCGCCCTCTT	ACTTCAGACTGGGGCTGC

n		
Drosocin	CCATCGTTTTCCTGCTGC	GCAGCTTGAGTCAGGTGA
Attacin A	ACAATCTGGATGCCAAGG	TCCCGTGAGATCCAAGGT
Diptericin	GTACTTGTTCGCCCTCTT	ACTTTCCAGCTCGGTTCT
Defensin	TTCTCGTGGCTATCGCTT	GGTGTGGTTCCAGTTCCA
Metchnikowin	TGCACTTAATCTTGGAG	ATCGAAAATGGGTCCCTG

Supplementary Table 3. Quantitation of crystal cell phenotypes in this study

Figure		*Genotype*	Crystal cell number (average±SD)	n	p value
**Fig.1**	b-e	*Oregon R*	50±27	20	
		$Gr63a^1$ (Outcrossed 50 generations)	115±59	51	<0.0001
		$Gr63a^1$ x Oregon R	125 ±67	22	<0.0001
	f-i	*Gr63a-gal4 x Oregon R*	87 ± 38	31	
		Gr21a RNAi x Oregon R	68 ± 27	20	
		UAS-hid,rpr x Oregon R	110 ± 33	20	
		UAS-Shi^{ts1} x Oregon R	60 ± 25	20	
		Gr63a-gal4 x Gr21a RNAi	289 ±113	22	<0.0001
		Gr63a-gal4 x UAS-hid,rpr	262 ±88	55	<0.0001
		Gr63a-gal4 x UAS-Shi^{ts1}	240 ±52	22	<0.0001
	j-l	*Gr63a-gal4 x Oregon R*	80 ±31	26	
		Gr21a RNAi x Oregon R	68 ± 27	20	
		Gr63a-gal4 x Gr21a RNAi	202 ±70	21	<0.0001
		(soda-lime) Gr63a-gal4 x Oregon R	160±85	37	<0.0001
		(soda-lime) Gr21a RNAi x Oregon R	136±65	20	<0.0001
		(soda-lime) Gr63a-gal4 x Gr21a RNAi	247±110	28	0.0008
**Supplementary Fig. 1**	d-f	*Oregon R*	50 ± 27	20	
		$Gr63a^1$ x Oregon R	125 ±67	22	<0.0001
		Df[Gr21a]/CyO,GFP x Oregon R	81 ± 34	27	0.0023
		Df[Gr21a]/ CyO,GFP x $Gr63a^1$	204 ± 55	22	<0.0001
	g-i	*Gr21a-gal4 x Oregon R*	82 ± 34	22	
		UAS-Shi^{ts1} x Oregon R	60±25	20	
		Gr21a-gal4 x UAS-Shi^{ts1}	204 ±55	25	<0.0001
	j-l	*HHLT-gal4 x Oregon R*	62 ±35	23	
		Gr21a RNAi x Oregon R	68 ± 27	20	
		HHLT-gal4 x Gr21a RNAi	84 ±37	32	0.0517
	u	*Elav-gal4 x Oregon R*	65 ± 31	22	
		Elav-gal4 x Gyc89da	183 ± 84	12	<0.0001

		RNAi			
**Fig.2**	a-d	*Gyc89da-gal4 x Oregon R*	88±32	52	
		UAS-NaChBac x Oregon R	73±38	21	
		UAS-Shi^{ts1} x Oregon R	60±25	20	
		Gyc89da-gal4 x UAS-Shi^{ts1}	96 ±38	20	0.5672
		(Shift) Gyc89da-gal4 x Oregon R	81 ± 48	37	
		(Shift) Gyc89da-gal4 x UAS-NaChBac	162 ±62	21	<0.0001
	e-g	*UAS-NaChBac x Oregon R*	73±38	21	
		Gyc89da-gal4; Elav-gal 80 x Oregon R	96 ±42	40	
		Gyc89da-gal4; Elav-gal 80 x UAS-NaChBac	99 ±36	35	0.9893
	h-i	*Gr63a-gal4; Gyc89da-gal4 x Oregon R*	121 ±27	36	
		UAS-Shi^{ts1} x Oregon R	60±25	20	
		Gr63a-gal4 x UAS-Shi^{ts1}	240 ±52		<0.0001
		Gr63a-gal4; Gyc89da-gal4 x UAS-Shi^{ts1}	110 ±35	33	<0.0001
	j-k	*Gyc89da-gal4 x Oregon R*	81 ± 48	37	
		UAS-Shi^{ts1} x Oregon R	60±25	20	
		Gyc89da-gal4 x UAS-Shi^{ts1}	95 ±31	24	0.0639
		(soda-lime) Gyc89da-gal4 x Oregon R	160±46	34	<0.0001
		(soda-lime) UAS-Shi^{ts1} x Oregon R	95±31	20	0.0003
		(soda-lime) Gyc89da-gal4 x UAS-Shi^{ts1}	62±39	27	<0.0001
	l	*Gr63a-gal4 x Oregon R*	87 ±38	31	
		Gad1 RNAi x Oregon R	50±17	20	
		Gr63a-gal4 x Gad1 RNAi	219 ±85	30	<0.0001
	m	*Gyc89da-gal4 x Oregon R*	88 ±32	52	
		$GABA_bR1$ RNAi x Oregon R	32 ± 17	20	
		$GABA_bR2$ RNAi x Oregon R	97 ± 45	20	
		Gyc89da-gal4 x $GABA_bR1$ RNAi	135 ±43	30	<0.0001
		Gyc89da-gal4 x $GABA_bR2$ RNAi	159 ±78	38	<0.0001
**Supplementary Fig. 2**	g	*Elav-gal4 x Oregon R*	65±31	22	
		Gyc89da-gal4 x Oregon R	88±32	52	
		UAS-sima RNAi x Oregon R	41±23	20	
		Elav-gal4; $Gr63a^1$	131±50	23	<0.0001
		UAS-sima RNAi; $Gr63a^1$	90±47	16	<0.0001
		Gyc89da-gal4; $Gr63a^1$	180±58	17	<0.0001

		Elav-gal4; Gr63a¹ x sima RNAi; Gr63a¹	43±21	20	<0.0001
		Gyc89da-gal4; Gr63a¹ x sima RNAi; Gr63a¹	34±20	11	<0.0001
**Fig.3**	g	*(Shift) Gyc89da-gal4 x Oregon R*	54 ±22	21	
		(Shift) Gyc89da-gal4 x UAS-sima	132±37	27	<0.0001
		(Shift) UAS-sima x Oregon R	43±8	20	
	h	*Gyc89da-gal4; Gr63a-LexA x Oregon R*	84 ±44	47	
		sima RNAi; LexAop-Shi^{ts1} x Oregon R	62±29	20	
		Gyc89da-gal4; Gr63a-LexA x LexAop-Shi^{ts1}	200±55	22	<0.0001
		Gyc89da-gal4; Gr63a-LexA x sima RNAi; LexAop-Shi^{ts1}	97 ±32	21	<0.0001
	i	*Gyc89da-gal4 x Oregon R*	81 ± 48	37	
		UAS-sima RNAi x Oregon R	41 ± 23	20	
		Gyc89da-gal4 x sima RNAi	86±27	18	0.2738
		(soda-lime) Gyc89da-gal4 x Oregon R	160±46	34	<0.0001
		(soda-lime) UAS-sima RNAi x Oregon R	146±35	20	<0.0001
		(soda-lime) Gyc89da-gal4 x sima RNAi	86±27	22	<0.0001
	j	*(Shift) Elav-gal4 x Oregon R*	73 ± 38	39	
		(Shift) UAS-sima x Oregon R	43±8	20	
		(Shift) Elav-gal4 x UAS-sima	138±59	20	<0.0001
	k	*Elav-gal4; Gr63a-LexA x Oregon R*	75 ±31	39	
		sima RNAi; LexAop-Shi^{ts1} x Oregon R	62±29	20	
		Elav-gal4; Gr63a-LexA x LexAop-Shi^{ts1}	166± 50	26	<0.0001
		Elav-gal4; Gr63a-LexA x sima RNAi; LexAop-Shi^{ts1}	64 ±28	22	<0.0001
**Supplementary Fig. 3**	e	*Oregon R*	50±27	20	
		upd3 Δ x Oregon R (male)	50±18	21	
		upd2 Δ upd3 Δ x Oregon R (male)	23±16	29	

		upd3 Δ x Gr63a^{1} (male)	80±65	34	0.0039
		upd2 Δ upd3 Δ x Gr63a^{1} (male)	47±19	26	<0.0001
		Gr63a^{1} x Oregon R	125±67	22	<0.0001
	f	*Elav-gal4 x Oregon R*	65±31	22	
		UAS-upd3 RNAi x Oregon R	88±41	20	
		UAS-upd3 RNAi; Gr63a^{1}	139±57	15	<0.0001
		Elav-gal4; Gr63a^{1}	131±50	23	<0.0001
		Elav-gal4; Gr63a^{1} x upd3RNAi; Gr63a^{1}	48±19	17	<0.0001
**Fig.4**	**c**	*(Shift) Elav-gal4 x Oregon R*	73 ± 38	39	
		(Shift) UAS-upd3 x Oregon R	87 ± 38	20	
		(Shift) Elav-gal4 x UAS-upd3	194±70	22	<0.0001
	d	*Elav-gal4 x Oregon R*	73 ±41	26	
		upd3RNAi x Oregon R	88 ± 41	20	
		Elav-gal4 x upd3 RNAi	86 ±42	24	0.3512
	e	*Elav-gal4; Gr63a-LexA x Oregon R*	75±31	21	
		upd3 RNAi; LexAop-Shi^{ts1} x Oregon R	69 ±41	20	
		Elav-gal4; Gr63a-LexA x LexAop-Shi^{ts1}	166±50	27	<0.0001
		Elav-gal4; Gr63a-LexA x upd3 RNAi; LexAop-Shi^{ts1}	104±48	24	<0.0001
	f	*Elav-gal4 x Oregon R*	73±41	26	
		upd3RNAi x Oregon R	75 ± 40	20	
		Elav-gal4 x upd3 RNAi	86±42	24	0.3512
		(soda-lime) Elav-gal4 x Oregon R	134±55	26	<0.0001
		(soda-lime) upd3RNAi x Oregon R	151±37	20	<0.0001
		(soda-lime) Elav-gal4 x upd3 RNAi	76±52	26	0.0012
	r	*ppl-gal4; Gr63a-LexA x Oregon R*	94±57	53	
		dome RNAi; LexAop-shi^{ts1} x Oregon R	89±37	20	
		ppl-gal4; Gr63a-LexA x LexAop-Shi^{ts1}	191±73	35	<0.0001
		ppl-gal4; Gr63a-LexA x dome RNAi; LexAop-Shi^{ts1}	76±31	22	<0.0001
	s	*ppl-gal4 x Oregon R*	26±19	27	
		ppl-gal4 x dome RNAi	45±16	20	0.0005

		(soda-lime) ppl-gal4 x Oregon R	107±59	22	<0.0001
		(soda-lime) dome RNAi x Oregon R	131±41	20	<0.0001
		(soda-lime) ppl-gal4 x dome RNAi	48±22	28	<0.0001
**Supplementary Fig. 4**	g	*Repo-gal4 x Oregon R*	72±17	17	
		UAS-dilp6 x Oregon R	112 ±62	21	0.0662
		Repo-gal4 x UAS-dilp6	107±50	42	0.0115
	h	*Oregon R*	50±27	20	
		$dilp6^{41}$ x Oregon R	40±20	20	0.2285
		$Gr63a^{1}$ x Oregon R	125±67	22	<0.0001
		$Dilp6^{41}$ x $Gr63a^{1}$	51±29	20	<0.0001
	k	*Repo-gal4 x Oregon R*	57±38	27	
		dilp6 RNAi x Oregon R	105±39	20	0.0003
		Repo-gal4 x dilp6 RNAi	78±29	21	0.0416
		(soda-lime) Repo-gal4 x Oregon R	116±52	38	<0.0001
		(soda-lime) dilp6 RNAi x Oregon R	148±30	20	<0.0001
		(soda-lime) Repo-gal4 x dilp6 RNAi	118±53	34	0.7866
**Fig.5**	e	*ppl-gal4 x Oregon R*	44 ±28	27	
		UAS-dilp6 x Oregon R	112 ±62	21	0.006
		ppl-gal4 x UAS-dilp6	169±57	17	<0.0001
	f	*ppl-gal4; Gr63a-LexA x Oregon R*	93±55	56	
		dilp6 RNAi; LexAop-Shi^{ts1} x Oregon R	98±37	20	
		ppl-gal4; Gr63a-LexA x LexAop-Shi^{ts1}	191±73	34	<0.0001
		ppl-gal4; Gr63a-LexA x dilp6 RNAi; LexAop-Shi^{ts1}	83±47	17	<0.0001
	g	*ppl-gal4 x Oregon R*	26±19	27	
		dilp6 RNAi x Oregon R	105±39	20	<0.0001
		ppl-gal4 x dilp6 RNAi	41±27	30	0.015
		(soda-lime) ppl-gal4 x Oregon R	107±59	22	<0.0001
		(soda-lime) dilp6 RNAi x Oregon R	148±30	20	<0.0001
		(soda-lime) ppl-gal4 x dilp6 RNAi	41±27	30	<0.0001
**Supplementary Fig. 5**	f	*Normoxia (Gr63a-gal4 x Oregon R)*	51±29	20	
		Hypercapnia (Gr63a-gal4 x Oregon R)	49±23	20	0.9138
	l	*Hml-gal4 x Oregon R*	115±43	46	

		Hml-gal4 x Serrate RNAi	25±18	28	<0.0001
**Fig.6**	b	*Gr63a-gal4 x Oregon R*	80±31	26	
		Gr21aRNAi x Oregon R	68 ± 27	20	
		Gr63a-gal4 x Gr21a RNAi	202±70	21	<0.0001
		(soda-lime) Gr63a-gal4 x Oregon R	160±85	37	<0.0001
		(soda-lime) Gr21a RNAi x Oregon R	136±65	20	<0.0001
		(soda-lime) Gr63a-gal4 x Gr21a RNAi	247±110	28	0.0004
	c	*Gr63a-gal4 x Oregon R*	80 ±31	26	
		Gr21aRNAi x Oregon R	68 ± 27	20	
		(Hypoxia) Gr63a-gal4 x Oregon R	174±63	13	<0.0001
		(Hypoxia) Gr63a-gal4 x Gr21a RNAi	144±49	11	0.1642
	d	*Gr63a-gal4 x Oregon R*	80 ±31	26	
		Gr21aRNAi x Oregon R	68 ± 27	20	
		Gr63a-gal4 x Gr21a RNAi	202 ±70	21	<0.0001
		(Hyperoxia) Gr63a-gal4 x Oregon R	39±20	11	0.0004
		(Hyperoxia) Gr63a-gal4 x Gr21a RNAi	88±37	12	0.0006
